# Assessing User Experience with Piezoresistive Force Sensors: Interpreting Button Press Impulse and Duration

**DOI:** 10.3390/s25216685

**Published:** 2025-11-01

**Authors:** Carlos Gilberto Gomez-Monroy, Vicente Borja, Alejandro Ramirez-Reivich, Maria del Pilar Corona-Lira

**Affiliations:** 1 Postgraduate School of Engineering, Universidad Nacional Autónoma de México, Ciudad Universitaria, Ciudad de México 04510, Mexico; 2 Department of Mechanical Engineering, Universidad Nacional Autónoma de México, Ciudad Universitaria, Ciudad de México 04510, Mexico; vicenteb@unam.mx (V.B.); areivich@unam.mx (A.R.-R.); pilicorona@gmail.com (M.d.P.C.-L.)

**Keywords:** user experience, human-robot interaction, force sensors, physical human-robot interaction, embedded UX, privacy-aware sensing, piezoresistive force sensors

## Abstract

As robotic systems become increasingly integrated into daily life, the need for user experience (UX) assessment methods that are both privacy-conscious and suitable for embedded hardware platforms has grown. Traditional UX evaluations relying on vision, audio, or lengthy questionnaires are often intrusive, computationally demanding, or impractical for low-power devices. In this study, we introduce a novel sensor-based method for assessing UX through direct physical interaction. We designed a robot lamp with a force-sensing button interface and conducted a user study involving controlled robot errors. Participants interacted with the lamp during a reading task and rated their UX on a 7-point Likert scale. Using force and time data from button presses, we correlated force and time data to user experience and demographic information. Our results demonstrate the potential of bodily interaction metrics as a viable alternative for UX assessment in human-robot interaction, enabling real-time, embedded, and privacy-aware evaluation of user satisfaction in robotic systems.

## 1. Introduction

Advances in sensor technology and embedded systems have profoundly shaped the way users interact with modern devices. From smartphones and wearables to autonomous vehicles and service robots, sensors now mediate nearly every aspect of human-device interaction. Among these, force sensors have traditionally served functional roles, enabling touch detection or object manipulation [1]. However, their potential to convey deeper information about the user, such as emotional state or level of engagement, remains underexplored [2,3].

As physical interaction continues to complement or even surpass visual and voice-based interfaces in various domains, understanding how users interact with devices (such as pressing, gripping, or handling them) may provide valuable insights into their experience [4,5,6,7]. This perspective aligns with recent efforts to design robots that are not only responsive to commands but also tailored to how those commands are delivered [4,8]. Within the context of data-driven robotics, interpreting physical human-robot interaction (HRI) as dynamic signals that contain traces of the user’s emotional state opens up new possibilities for implementing off-the-shelf sensors [9,10].

For instance, Hayashi et al. [11] conducted an experiment on touch communication between humans and robots by adding touch sensors to the robot. They then related the collected data from the users’ physical interaction with the robot to the users’ subjective evaluations of such an interaction. A second example is Ghosh et al. [12] who established a connection between sensor-based interaction data and subjective assessment through psychometric modeling by instrumenting shoes with an array of thin resistive force sensors; the embedded sensors captured fine-grained physical traces (such as gait dynamics and pressure distributions) from users wearing the instrumented footwear, and the data were then correlated with extensive self-reports (133 questionnaire items). And a third example is the study by Bilyea et al. [13], in which the authors used three robotic tools for daily living activities (hair brushing, face wiping, and face shaving) to study the forces volunteers apply during use; the purpose of the study was to characterize the subjective user state of “keeping a comfortable force level” into sensor-based metrics (force measurements). These three examples demonstrate how embodied sensing can reveal subjective details of the user’s perception.

There is extensive research on sensor technologies that enable complex human-system interactions, a significant portion of which focuses on optical and audio applications (consistent with the rise in cameras and mics embedded in robots); nevertheless, research on force-sensing technologies for physical human-robot interactions (pHRI) remains underrepresented in the field.

In this regard, our research question regarding force-sensing technologies for pHRI is the following: do the force and duration of the user’s button pushes provide information about the user’s physical characteristics and emotional states?

In this paper, we report how off-the-shelf piezoresistive force sensors can be utilized to scrutinize the user’s objective and subjective experiences when embedded in interactive robots. In practice, we explored the capacity of force sensors to serve as indicators of the user’s mental state by focusing on how users pushed the buttons of a robot lamp in five different experimental tests. The robotic lamp was programmed to behave differently in each test to compare the data from the user’s button-pushing behavior (force and duration of the pushes) with the user’s self-evaluated user experience (UX) and demographic information.

**Figure 1:**
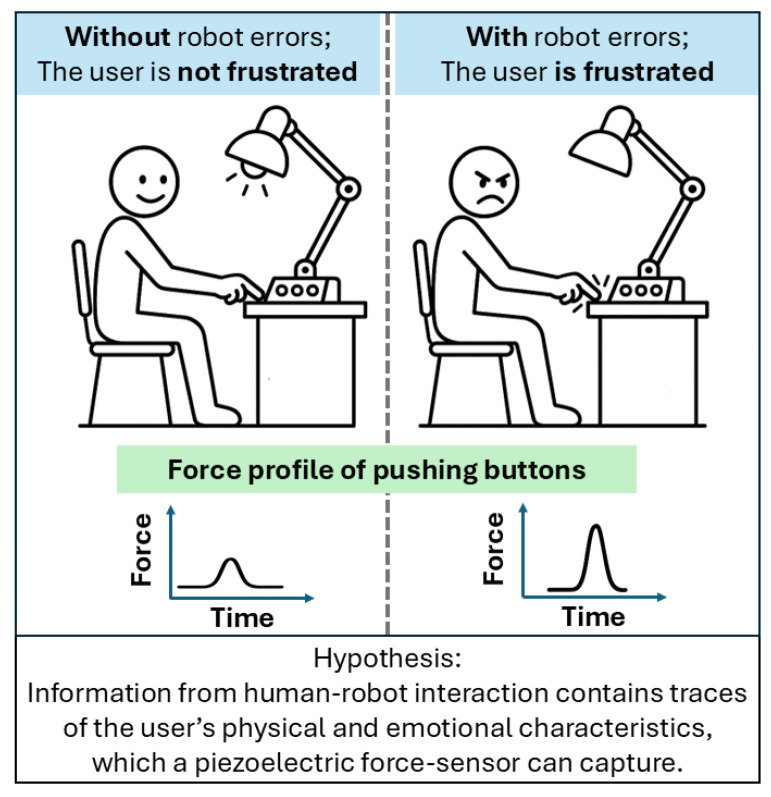
Users push buttons harder in the robot’s interface when users are frustrated by robot errors.

In other words, our study focuses on analyzing the users’ button-pressing behaviors observed during interactions with a robot lamp, and we provide evidence that cumulative force–time data correlates with the users’ UX [14,15]. The user study was designed to test if users would express their frustration towards robot errors in a bodily and physical way, by pushing the buttons harder when the robot malfunctions, see Figure 1.

Our results show that, in fact, higher error rates would reduce UX ratings and increase the intensity or duration of physical interaction. This may be somewhat expected; nevertheless, the novel contribution of our study lies in establishing a connection between subjective evaluations and interaction data obtained with force-sensors during the HRI.

Notably, the use of piezoresistive force sensors enables not only the analysis of physical input behaviors (such as the strength and duration of button presses) but also the examination of how these physical interactions may correlate with user demographics, including weight, age, and gender. The integration of force-sensors and data-driven robotics demonstrates the broader potential of sensor-based approaches for capturing both behavioral and individual differences in human-robot interaction.

The remainder of this paper presents the state-of-the-art, the method and materials of the experimental setup, the results, and the discussion of implementing force sensors in interactive systems.

## 2. State-of-the-Art

### 2.1. UX Evaluation in Human-Robot Interaction

User experience (UX) evaluation has become a central aspect of assessing the quality of human-robot interaction (HRI). Early frameworks stressed holistic perspectives that capture both cognitive and affective elements of user responses during interactions [16]. However, traditional methods such as self-report questionnaires, interviews, or emotion recognition systems remain limited by intrusiveness, cognitive burden, and their reliance on external devices or resource-intensive computation [2,17]. Recent systematic reviews have highlighted the challenge of aligning evaluation methods across laboratories and application domains, noting inconsistencies in how UX is conceptualized and measured [18]. These limitations motivate the search for lightweight, scalable, and replicable methods that can be deployed in embedded robotic platforms.

### 2.2. Written Questionnaires in UX Assessment

Questionnaires remain one of the most widely used tools for UX evaluation in both human-computer and human-robot interaction. Instruments such as the AttrakDiff questionnaire [19], and the User Experience Questionnaire (UEQ) [20] offer structured methods for capturing user perceptions. Their adoption across domains has established benchmarks for usability and experience measurement, and their psychometric properties are well documented [21,22]. However, the repeated administration of lengthy questionnaires can impose cognitive fatigue, disrupt the flow of interaction, and reduce ecological validity in both short and embedded interactions [23]. In robotic contexts, these issues are particularly problematic when evaluations must be performed during ongoing activities or on low-power platforms without external supervision. Recent discussions emphasize the need for lightweight alternatives that preserve the richness of UX measurement while minimizing user burden [17,18]. This creates a gap for methods that maintain the validity of UX constructs but rely on more direct and unobtrusive data sources.

### 2.3. Sensor-Based Approaches to UX and Interaction

Advances in tactile, haptic, and force-sensing technologies have created opportunities for the unobtrusive evaluation of user experience. Force and pressure sensors are widely applied in rehabilitation devices [24], wearable systems for collaborative play [25], and robotic exoskeletons [26]. Reviews confirm that force-sensitive interface engineering is a rapidly growing field, enabling the high-resolution capture of bodily interaction metrics relevant for HRI [27,28]. Computational methods have also been developed to process tactile data streams, including machine learning pipelines that link sensor signals to interaction outcomes [29,30]. These works demonstrate the feasibility of leveraging bodily interaction for assessing user states, though their application to UX assessment in privacy-sensitive contexts is still emerging.

### 2.4. UX Evaluation Frameworks in Embedded and Privacy-Aware Systems

Several recent studies emphasize the need for UX methods that are not only accurate but also privacy-respecting and suitable for resource-constrained devices. For instance, wearable and ambient intelligence systems increasingly integrate UX sensing into everyday contexts [31,32]. Embedded devices with tactile or force-based interfaces provide a unique opportunity to bypass vision and audio sensing, thus avoiding privacy concerns while reducing computational demands [33]. Novel approaches include multimodal log-based UX evaluation [34] and hybrid frameworks that combine implicit sensor-based measures with explicit feedback [12,13]. These directions align with the development of real-time, embedded UX assessments deployable in mechanical systems and consumer robotics.

### 2.5. Gap and Contribution

Despite these advances, there remains limited research on the implementation of force-sensing robot capabilities for direct and physical human-robot interaction. There is a gap in the assessment of bodily mediated HRI focused on direct physical interaction metrics, such as force and time. Prior works often combine multiple modalities or rely on computationally demanding pipelines [35]. The present study contributes to this gap by demonstrating that simple bodily interaction data (i.e., force and duration of button presses) can serve as proxies for subjective UX ratings, as sensor-based data correlates with self-reported UX. Moreover, this work provides empirical evidence that privacy-conscious, embedded, and time-efficient UX evaluation is feasible (as an alternative to camera and mic technologies).

## 3. Methods

### 3.1. Participants and Experimental Design

Forty-six individuals (30% female, 70% male; age range = 18–68 years, *M* = 32) were recruited from the School of Engineering at the National Autonomous University of Mexico (UNAM). Participation was voluntary and uncompensated. Participants were randomly assigned to one of three experimental groups, defined by the initial lamp height (15, 30, or 45 cm), to examine whether the starting configuration influenced user experience (UX). Instead of a control group, we employed a test–retest design with two zero-error trials (Tests 1 and 4) providing internal reliability checks. All procedures complied with institutional ethical standards: participants signed informed consent forms, anonymized data were collected using robot-generated identifiers, and all participants were debriefed upon completion.

### 3.2. Materials: The Robot Lamp

We chose to design a robot lamp because there is an interest in the automation of human living spaces. Part of such automation is the development of robotic appliances that handle comfort settings and even assist people with specific demands (e.g., the elderly or autistic people) [31]. The robot lamp was designed for experimental control and fine-grained data acquisition. It comprised five adjustable parameters: height (15/45 cm, above desk), brightness (50/1200 lux), projection angle (0°/−160°), button sensitivity (20/1200 gr), and ambient illumination (only as “On” or “Off”). Participants directly manipulated the height, brightness, and projection angle via a button-based interface, while button sensitivity and ambient lighting were automatically adjusted according to programmed interaction modes across trials.

From a technical perspective, height and projection angle functioned as mechanical degrees of freedom, while brightness, button sensitivity, and ambient illumination were interaction-level parameters. Although non-kinematic, these variables shaped UX and allowed systematic modulation within HRI research.

**Figure 2:**
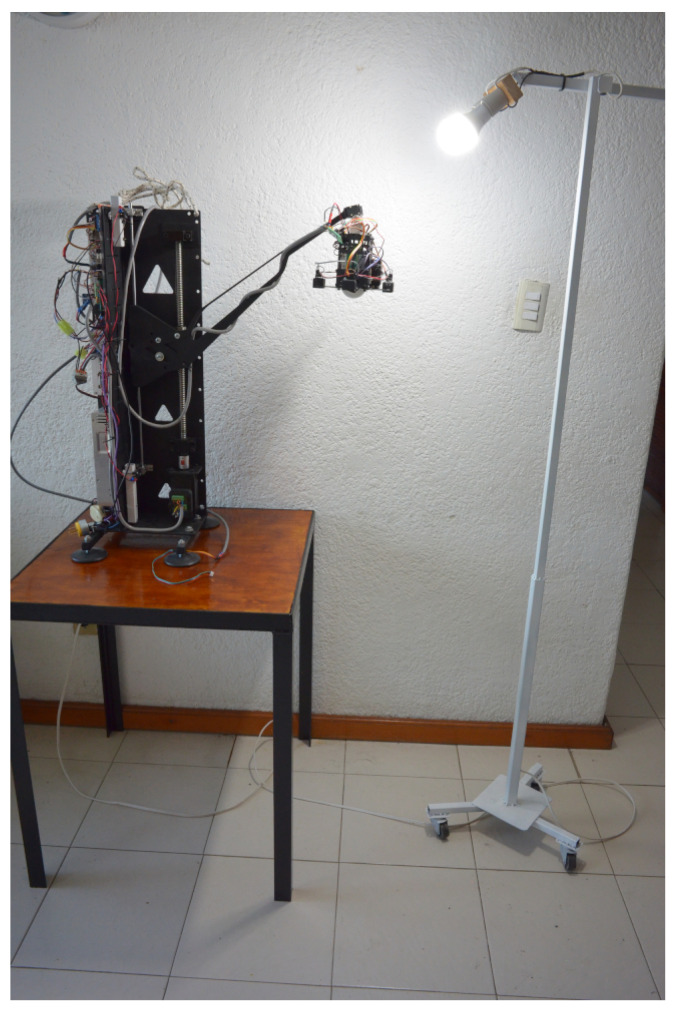
Robot lamp on desktop.

The robot lamp was implemented as a data-driven robotic platform, equipped with multiple processors (one Arduino Mega and two Arduino Unos) operating in parallel to support distributed task execution. A real-time clock (RTC) module and SD card module provided time-synchronized data storage, enabling the continuous logging of interaction variables. The system integrated four light sensors, two push buttons, and a master power switch. Actuation was achieved through a servo-stepper, two servos, and three high-intensity LED modules (5 W, 110 V, 5500 K). This architecture ensured parallel processing, real-time data acquisition, and reliable storage, establishing the lamp as a fully self-contained platform for human-robot interaction studies. This self-contained platform enabled high-fidelity data collection without the need for external sensors (Figure 2).

#### 3.2.1. Force Sensing

Button interactions were mediated by Honeywell FSG15N1A piezoresistive force sensors, rated up to 15 N with a sensitivity of 0.24 mV/g. These sensors were selected for their precision, linearity, and extremely low deflection (∼30 µm), which minimize tactile or auditory cues typically associated with conventional buttons. This ensured that robotic responses, rather than mechanical feedback, served as the primary perceptual cue of effective HRI.

**Figure 3:**
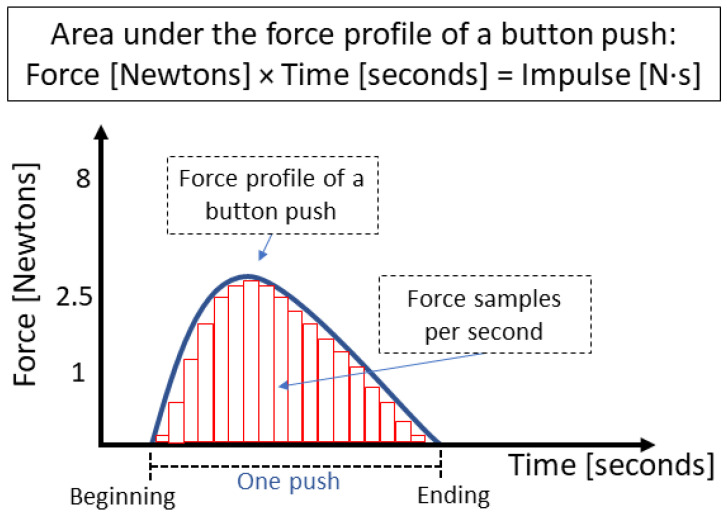
The blue line represents the force profile of a button press. The continuous curve represents the applied force over time, while the vertical bars (in red) indicate discrete force samples taken per second. The area under the curve corresponds to the mechanical impulse (Force×Time), capturing the total effect of the interaction.

Figure 3 illustrates the force profile of a button press, represented as a function of time. The blue profile depicts the dynamics of force applied during a single button press, while the red vertical bars indicate discrete force samples captured per second. The area under the curve, corresponding to the integral of force over time, represents the mechanical impulse delivered during the interaction. The diagram illustrates the temporal boundaries of a complete button press, from onset to release. It emphasizes that we calculated the impulse as the product of force (in Newtons) and time (in seconds).

Moreover, we selected piezoresistive force sensors with minimal plunger travel to ensure that HRI feedback was exclusively mediated through robotic responses, rather than by a “click” sound or the considerable travel of the sensor plunger. Therefore, we chose the Honeywell FSG15N1A sensors, which avoid both audible click sounds and haptic feedback, ensuring that the only perceptible cue for participants was the robot’s action upon reaching the force threshold.

**Figure 4:**
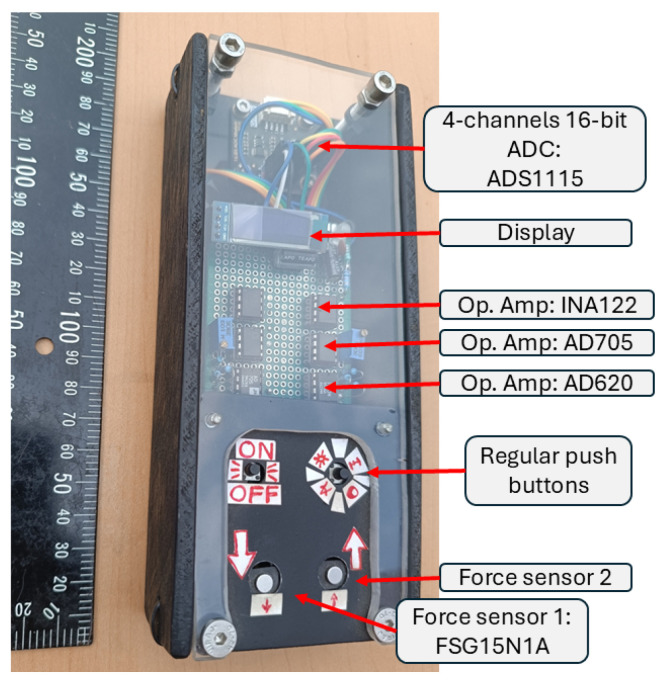
Robot-lamp interface. Sensing, analog signal processing, and digital conversion of the data inside the robot interface to minimize noise.

**Figure 5:**
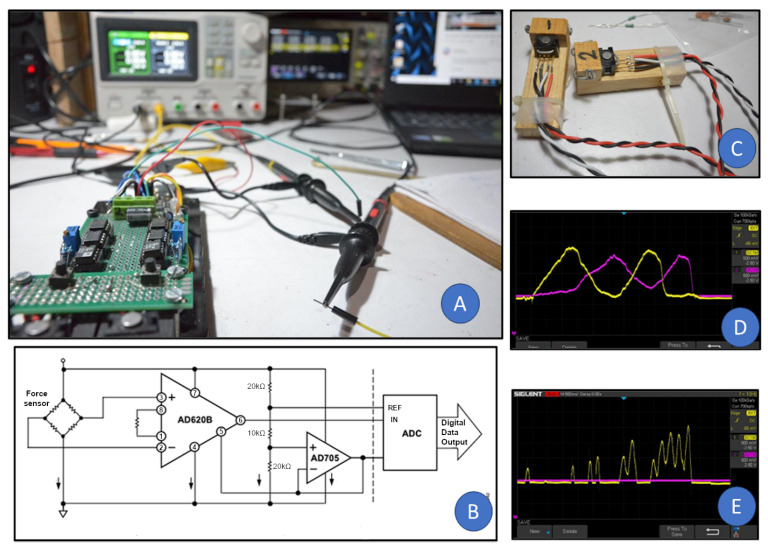
(**A**) Lab testing of the force sensors coupled to a chain of Op. Amp. (instrumental amplifiers) and a 16-bit ADC, (**B**) base electric circuit for the Op. Amp. AD620 and resistive bridge, (**C**) piezoresistive force sensors, Honeywell FSG15N1A, wired and set on their holders, (**D**) oscilloscope image of the two force sensors being pushed at the same time without issues in the rail-to-rail amplified signal, (**E**) oscilloscope image of one force sensor being pushed at a time without affecting the stability of the signal from the second sensor.

Signal conditioning employed a multi-stage amplification system (AD620, AD705, INA122) and digitization via a 16-bit ADS1115 analog-to-digital converter (ADC), yielding a resolution of ∼0.0026 N/count. This configuration reliably detected subtle variations in applied force (Figure 4 and Figure 5). The analog-to-digital conversion was set at 10 SPS, without digital filtering, and the impulse was calculated with a rectangular integration. The total gain of the operational amplifiers was 65, and they were configured as follows to achieve rail-to-rail amplification: the AD620’s gain was set to 13 and was not referenced to ground; the AD705’s gain was set to 1 and referenced the signal to ground; and the INA122’s gain was set to 5. Furthermore, we implemented a software debounce time of 20 ms in thresholding, and minimized hysteresis in the signal path by avoiding capacitive filtering and maintaining low inductive loads. Robot actions were triggered by a simple conditional threshold “if(force>threshold);robot action”.

#### 3.2.2. Force Sensor Characterization

The Honeywell FSG15N1A sensors were calibrated with reference weights ranging from 1 g to 1100 g (0.01 N to 10.78 N) to map applied force to digital counts and compare the readings from the left and right buttons.

Table 1 shows that the “left” sensor reliably detected forces as low as 1 g; on the other hand, the “right” sensor has an initial sensitivity of 20 g (when it starts providing digital readings). Beyond 20 g, both sensors behaved similarly, maintaining a consistent analog-to-digital conversion factor of 3–4 digital counts per gram. At higher forces, the left button reported slightly higher values than the right one, but both exhibited a systematic, nonlinear increase above 7 N. While these disparities limit absolute accuracy at higher loads, the results are predictable and consistent across sensors. We test the sensors by connecting them to each other’s signal processing channel; the variance followed the sensors, indicating that the variation is in the sensors and not in the signal processing circuitry.

**Figure 6:**
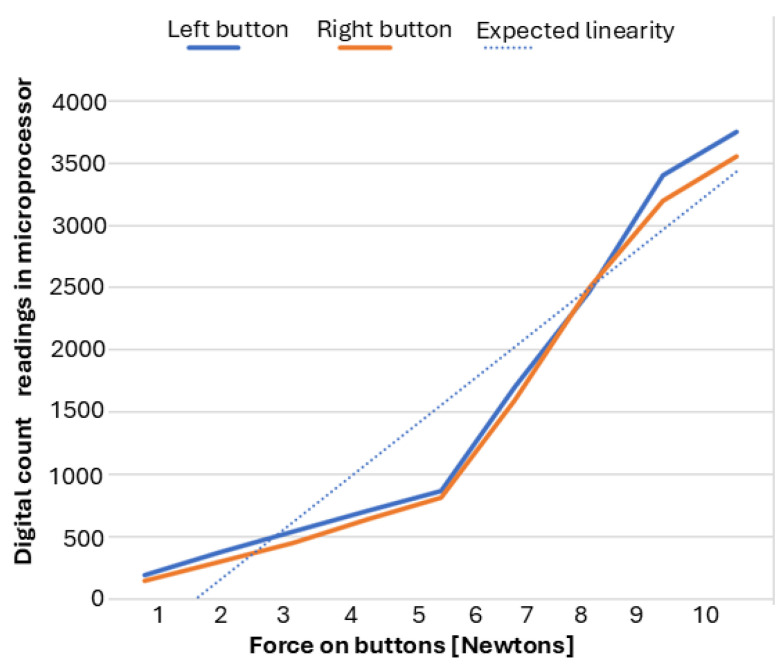
Plot of the characterization of both force sensors in relation to the digital counts logged by the microprocessor. The resulting trend lines portray the differences between sensors; nevertheless, both sensors showed a similar sigmoid-shaped response around the expected linearity.

Although the two force sensors exhibit slight differences in sensitivity and characterization, these variations remain consistent across all five tests and participants. Consequently, they are not considered a source of experimental variability. To preserve data integrity, the signals were not normalized via software, as both sensors operate within their specified ranges and demonstrated consistent repeatability during testing. Figure 6 illustrates the relationship between measured values and expected linearity, revealing a comparable sigmoid response in both sensors. Therefore, no correction was deemed necessary to compensate for differences between sensors.

### 3.3. Procedure

**Figure 7:**
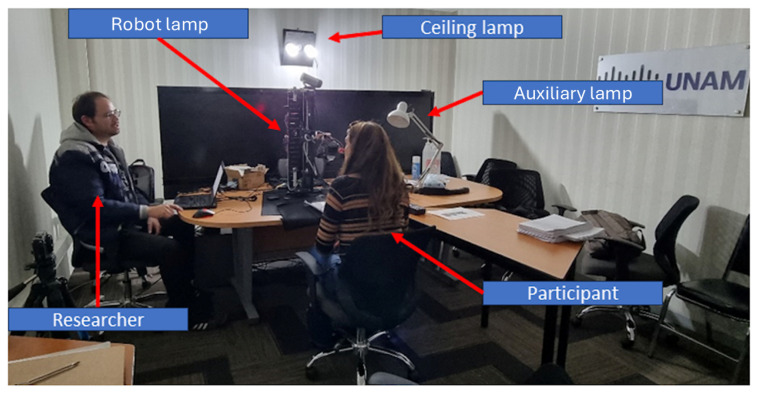
Photo of the user study setting showing the disposition of the participant, the interface and the robot lamp.

**Figure 8:**
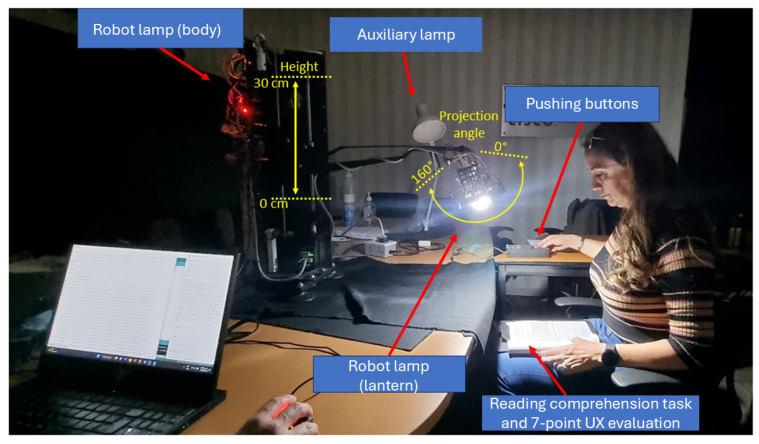
Photo of the user study showing the range of motion of the robot lamp.

Participants were seated in a windowless video-conference room, with the robot lamp positioned in front of them and a control panel to the right (Figure 7 and Figure 8). Using the robot lamp interface, they adjusted the lamp settings (height, projection angle, and brightness) to achieve the preferred lighting for a reading and writing comprehension task and UX evaluation. Each participant repeated this cycle five times, with the robot lamp behaving slightly differently each time by enacting robot errors and changing its operation mode (from manual to assisted). During the experimental tests, the ceiling lamp and auxiliary lamp remained turned off. The robot’s interface was on the right side of the participants, meaning that all participants used their right hand regardless of their dominant hand. Additionally, hand and finger size were not measured.

Robot behavior varied systematically across trials:

- Test 1: Zero errors (baseline).
- Test 2: Moderate error rate (25 malfunctions).
- Test 3: High error rate (75 malfunctions).
- Test 4: Zero errors (test–retest).
- Test 5: Distinct error mode (variable error distribution).

Five error types were introduced to emulate real-world malfunctions: (1) unrecognized input: the robot does not respond, (2) incorrect action: the robot does the opposite, (3) spontaneous activation: the robot moves by itself, (4) altered button sensitivity: user needs to push harder to trigger robot response, and (5) repeated action: “stuck button”. After each trial, the lamp reset to its default configuration and position while a secondary fixed lamp provided stable baseline illumination.

Every error was automatically generated and recorded by the RoLiS system, with no direct involvement from the researcher throughout the experimental sessions, with the triggers listed below:

- Signal Not Recognized: Valid presses are ignored.
  - Trigger type: Number of push
  - Test 2: 8, 9, 10, 11 and 12
  - Test 3: 12, 13, 14, 15, 16, 42, 43, 44, 45, 46, 62, 63, 64, 65, and 66
- Wrong Signal: The opposite command is executed.
  - Trigger type: Number of push
  - Test 2: 15, 18, 22, 32, and 45
  - Test 3: 11, 18, 19, 35, 39, 50, 55, 61, 67, 71, 79, 83, 85, 88, and 93
- Signal Stuck: The last action repeats as if a button is stuck.
  - Trigger type: Number of push
  - Test 2: 3, 17, 20, 35 and 40
  - Test 3: 2, 8, 10, 20, 38, 40, 54, 69, 68, 73, 78, 81, 86, 90, and 95
- Ghost Signal: The lamp moves without user input.
  - Trigger type: Time since last push
  - Test 2: 25 s (max. 5 errors)
  - Test 3: 15 s (max. 15 errors)
- Reduced Signal Sensitivity: Stronger force required to activate.
  - Trigger type: Number of push
  - Test 2: 26, 27, 28, 29, and 30
  - Test 3: 3, 4, 5, 6, 7, 21, 22, 23, 24, 25, 56, 57, 58, 59, and 60

### 3.4. Measures

UX was evaluated using a stimulus-response framework. The programmed robot behavior (stimulus) was defined by interaction mode and error rate. Participant responses were assessed on two levels:

1. Subjective: After each test, participants rated their experience on a single-item 7-point Likert scale. This minimized cognitive load and task interruption.
2. Objective: Sensor data included button press force, press duration, real-time values for lamp height, brightness, projection angle, and robot states (e.g., height, projection angle, and brightness).

## 4. Results

We begin this section by assessing the impact of the two experimental variables. One is the robot behavior (number of robot errors and operation mode) determined by experimental tests. The other is the robot lamp’s initial position, which was different for the three experimental groups.

Subsequently, we share the correlation matrix for sensor-based interaction variables (time and impulse spent pushing buttons), subjective evaluation of the HRI (self-reported UX), and demographic information of the participants (age, weight, and gender).

Thereafter, the analysis of the UX for such a test is shown (averaged and subdivided by demographic variables). Finally, we present the study of the time and the impulse spent pushing buttons (including a subdivision by demographic variables).

### 4.1. Impact of the Robot Lamp’s Initial Position and Behavior

The analysis of user experience (UX) scores across the three experimental groups was conducted using ANOVA. Results indicated that the differences between groups were not statistically significant, F = 1.97, *p* = 0.143. These findings suggest that group assignment (i.e., the robot’s initial position) did not produce meaningful variation in reported UX outcomes. In contrast, when comparing UX scores across the five experimental tests, ANOVA revealed a significant effect, F = 9.34, *p* < 0.001. This result indicates that participants’ UX ratings varied meaningfully across different experimental tests, suggesting that test conditions (robot behavior) influenced the user experience.

Furthermore, we conducted an omnibus test to analyze the overall impact of the test condition (changes in the robot lamp behavior) on Time and Impulse during direct button-pushing when interacting with the robot lamp, as well as on self-reported UX ratings, see Table 2. The values for Time, F = 2.13, *p* = 0.081, $\eta_{p}^{2}=0.06$, indicated that the test conditions did not reliably alter Time; at most, it shows a modest variability. Sphericity was evaluated via Mauchly’s test, and Greenhouse–Geisser (GG) correction was applied; this confirms the significance of the effect of the impulse and UX evaluations.

Noticeably, significant effects were found for both impulse and UX. Impulse revealed a significant effect associated with the experimental test, F = 10.52, *p* < 0.001, $\eta_{p}^{2}=0.25$. At the same time, UX demonstrated an even more substantial effect, F = 12.80, *p* < 0.001, $\eta_{p}^{2}=0.29$. These findings indicate that the test manipulation (different robot behaviors) produced substantial changes in these outcomes, with large effect sizes supporting the robustness of the observed differences.

### 4.2. Correlation Matrix: Relation Between Experimental and Demographic Data

To further examine the relationship between participants’ direct interactions with the robot lamp and their subjective evaluations, a correlation analyses was conducted. This analyses enabled the identification of potential associations between interaction effort (measured by the time and impulse spent pushing buttons) and demographic variables (age, gender, weight, and height), as well as self-reported user experience (assessed through a 7-point Likert scale), across the five experimental conditions.

At the individual level (see Table 3) correlations are weaker and dispersed, as expected due to variability in personal interaction styles and evaluations. The relation between UX and button interaction effort is negative (time: r = −0.13; impulse: r = −0.33), hinting that participants who engaged with less physical intensity tended to evaluate the experience more positively. Demographic factors such as age, weight, and height showed negligible associations with UX (all with a correlation coefficient of approximately r = −0.10). This underscores the importance of considering personalized patterns of human-robot interaction.

### 4.3. Plotting UX: Effect of Robot Behavior and Demographics

**Figure 9:**
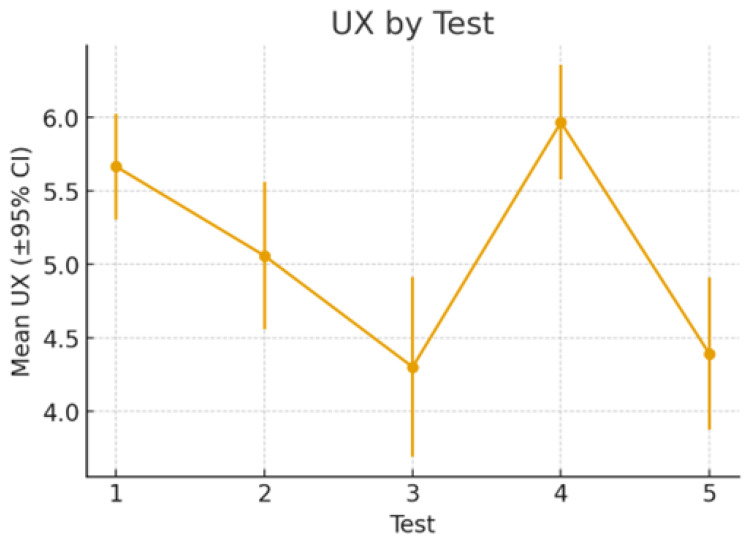
Mean user experience (UX) scores across five test sessions with 95% confidence intervals.

We illustrate the progression of the mean user experience (UX) ratings across the five testing sessions in Figure 9. The results reveal an initial decline in UX from Test 1 (M≈5.6) through Test 3 (M≈4.3), followed by a substantial improvement in Test 4 (M≈6.0). However, UX decreased again at Test 5 (M≈4.4). The error bars show that confidence intervals overlap in several cases, indicating that some differences may not be statistically significant. While statistical significance at the individual level is noisy, averaging across participants in the group smooths out this variability, allowing clearer trends to emerge. Nevertheless, peak UX evaluations occurred at Test 1 and Test 4 in which the robot made no errors (therefore, generating a positive experience); on the other hand, Test 3 (the test with the most robot errors) and Test 5 (the test in which the robot lamp changed its operation mode without letting the user know) received the worst UX evaluations (therefore, generating a frustrating experience).

**Figure 10:**
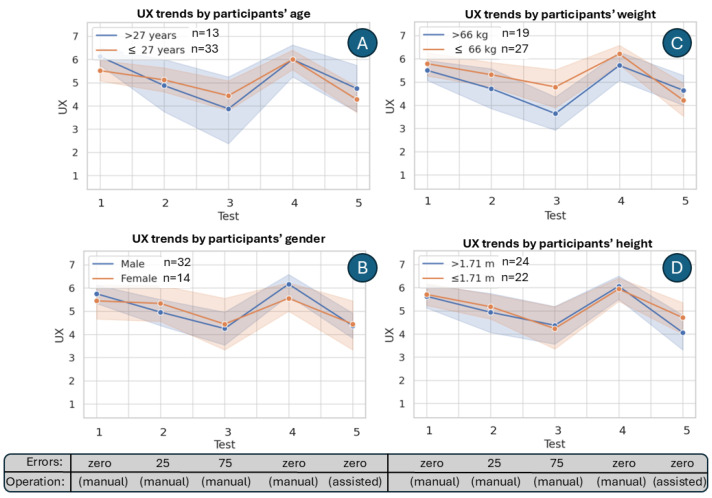
Comparison of UX ratings across five tests grouped by the participants’ demographic information (**A**), age (**B**), gender (**C**), weight, and (**D**) height. Shaded areas represent 95% confidence intervals to visualize uncertainty around the mean trends.

When stratifying the results by demographic groups, a consistent pattern of UX evaluation remains; as shown in Figure 10, all groups show a drop in UX ratings up to Test 3, followed by a marked peak at Test 4, and a subsequent decline at Test 5. Demographic differences, however, highlight moderating effects. Younger participants (≤27 years) and lighter individuals (≤66 kg) generally reported higher UX, particularly at Test 4, whereas older and heavier participants showed less variability between tests. Gender-based differences were less pronounced, though male participants exhibited slightly higher peaks. In terms of height, there is no substantial difference, as predicted by the correlation matrix’s value corresponding to the correlation between the UX and the height of the participants. These findings suggest that demographic factors influence the magnitude of UX ratings but do not alter the underlying progression across tests. We stratified our sample information as “above the mean” or “below the mean” per demographic characteristic; in other words, 27 years was the mean age for participants; similarly, 66 kg and 1.71 m were the mean weight and height of the sample.

### 4.4. Plotting Duration and Impulse: Effect of the Robot Behavior on Button Pushing

**Figure 11:**
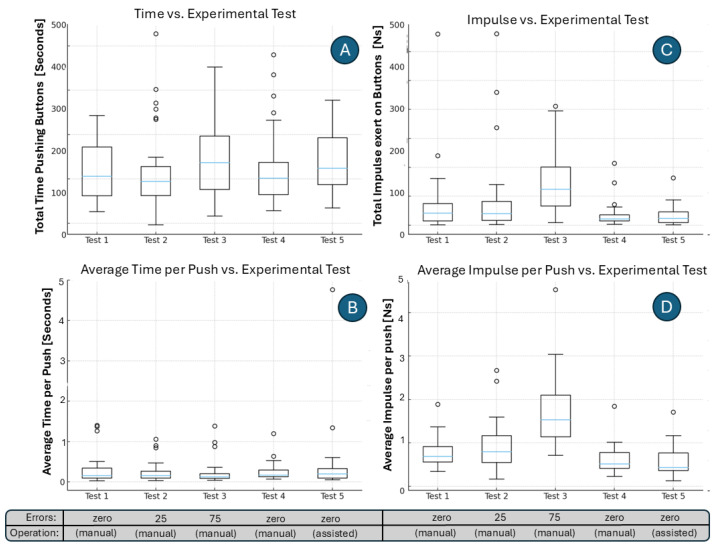
Boxplots of interaction metrics across five testing sessions. (**A**) Time spent pushing the force-sensing buttons of the robot lamp. (**B**) Average time per push. (**C**) Impulse (force applied multiplied by push duration). (**D**) Average impulse per push.

In Figure 11, we present boxplots of user interaction metrics across five testing sessions. Plot A reports the total time volunteers spent pushing the force-sensing buttons of the robot lamp. While overall times fluctuate across tests, the median duration decreases slightly in Test 4 compared to Test 3 and Test 5, suggesting a temporary increase in efficiency. Plot B shows the average time per push, where median values remain relatively stable. Plot C depicts impulse values; it shows substantial dispersion in Test 3, while Test 4 demonstrates lower and more consistent impulses. Plot D presents the average impulse per push, again revealing variability across tests, with Test 3 producing the highest medians and spreads. Taken together, these results indicate that although individual-level data is noisy, group-level averages smooth variability and highlight broader performance patterns, most notably the reduction in both time and impulse observed in Test 4 compared to other sessions.

### 4.5. Subdiving Force-Sensor Data by Demographic Characteristics

**Figure 12:**
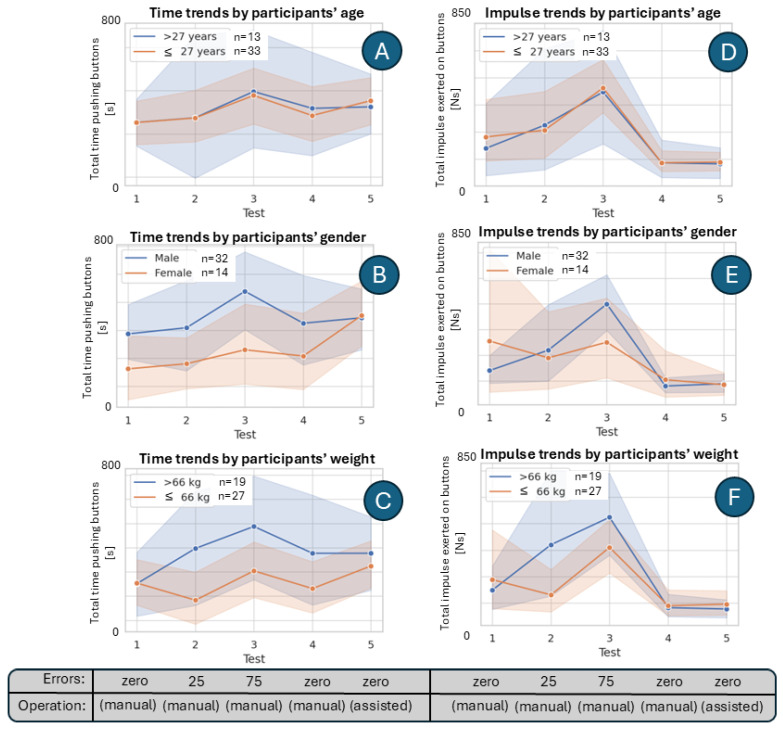
Interaction of test sessions with age and weight groups. Plots (**A**,**B**) show average time per push, and Plots (**C**,**D**) show average impulse per push, highlighting differences between participant groups across tests.

In Figure 12, we present the interaction between test sessions and participant characteristics (age, gender, and weight) in terms of time and impulse. Plot A shows the interaction between test and age group with respect to time, where older participants (>27 years) generally spent more time per push than younger participants (≤27 years). Plot B illustrates the interaction between test and gender group with respect to time, indicating that men consistently spent more time per push than women across all sessions, particularly in Test 3 where the gap peaked. Plot C displays the interaction between test and weight group with respect to time, showing that heavier participants (>66 kg) exhibited longer pushing times than lighter participants, especially in Tests 2 and 3.

Plot D reports the interaction between test and age group with respect to impulse, where both age groups followed similar trajectories, peaking at Test 3. Plot E highlights the interaction between test and gender group for impulse, demonstrating that men produced higher impulses than women when challenged with robot errors, with Test 3 again marking the most substantial divergence. Finally, Plot F presents the interaction between test and weight group for impulse, revealing that heavier participants generated substantially greater impulses compared to lighter ones, particularly in Tests 2 and 3.

Together, these findings suggest that participant characteristics (most notably weight and gender) influenced both the duration and strength of pushes, with differences becoming most evident in the third test session.

## 5. Discussion

In our robot lamp, piezoresistive force-sensors were implemented as buttons in the robot interface. Such force-sensitive buttons delivered reliable force sensing, detecting the push of a button with a resolution of 3 digital counts per gram, and remaining quasi-linear up to 10 N (Table 1). This performance profile is well-aligned with the role of tactile sensing in HRI pipelines reported in the field [36] and supports richer inferences through embodied signals [37,38].

Across the different testing conditions, consistent trends emerged in participants’ interactions with the robot lamp. In manual mode without programmed errors (Tests 1 and 4), users exhibited shorter interaction times and lower impulses, reflecting more efficient and less forceful button presses. In contrast, the introduction of robot errors in manual mode (Tests 2 and 3) led to pronounced increases in both interaction time and applied impulse, with Test 3 (characterized by 75 robot errors programmed) yielding the longest interaction times and strongest impulses, underscoring the burden of error correction. The assisted mode (Test 5) produced intermediate outcomes: while interaction times increased for some participants, impulses decreased relative to error-heavy manual sessions. However, it seems that participants often interpreted the assistance as unexpected robot errors, reporting lower UX similar to Test 3 manual sessions (the one with the most robot errors), which highlights critical implications for usability and design [39]. Demographic analyses further indicated that heavier and older participants pressed longer and harder, while men applied longer, stronger pushes, particularly under error-heavy conditions (Test 3 in Figure 12). Furthermore, individual-level associations are present but weak (UX–time: r = −0.13; UX-impulse: r = −0.33; Table 3), reflecting personal differences in interaction preferences.

It is worth noticing that our participant sample presented gender imbalances, which may influence the generalizability of the results. Although our pool included both male and female participants, the distribution was uneven (30% female, 70% male). This imbalance limits the extent to which gender-related patterns can be confidently generalized to broader populations. Nonetheless, the inclusion of 14 female participants remains acceptable for an exploratory study, as similar sample sizes are reported in HRI research [40,41].

The approach used in this research can be extended beyond buttons to encompass other robot parts whose primary functions are not typically associated with interaction, such as arms, joints, or even the robot’s surface materials. These elements can be designed to both actuate and sense, enabling the robot to interpret subtle user cues through touch, pressure, or motion [42]. For instance, a robot arm could measure resistance or compliance during a shared manipulation task, or a flexible shell could detect contact distribution and temperature variations [43]. By embedding sensing capabilities into such structural components, the robot’s body itself becomes an interface, allowing richer, more embodied communication between human and machine [44].

For future work, our empirical results motivate and support the use of tactile sensing as a practical channel for on-board estimation of user affective states. As data-driven HRI becomes more prevalent, personal attributes such as age, gender, weight, and height will play an increasingly important role in providing tailored user experiences and ensuring the user’s well-being in long-term HRIs [45,46]. Therefore, exploring approaches that link sensor-based data with demographic information in HRI is essential for developing more inclusive, adaptive multimodal human-robot interactions [47,48].

In summary, in this work, we demonstrated that force-sensing buttons provide high resolution for subtle interactions and cues of the subjective user experience. Future work will expand these findings by leveraging predictable sensor characteristics and behavior-linked metrics (number of pushes, time and force of pushing buttons, and UX), robots can modulate assistance and error handling to reduce effort and improve experience, moving beyond command recognition toward physically grounded, empathic interaction [49,50].

Here is a short insight regarding building interactive robots. Robot interfaces must remain clear and intuitive so that a limited set of sensors can capture meaningful information about the user. Our user study demonstrates that frustration arising from usability issues or robotic errors is reflected in measurable variations in interaction force and time. These metrics serve as objective indicators of user experience, enabling continuous monitoring and potentially reducing reliance on post-interaction surveys while moving toward real-time UX assessment. Equally important, analog signal processing should be minimized by converting data to digital form as early as possible and thoroughly characterizing every component, since even high-precision systems exhibit performance fluctuations that can affect interaction stability.

## 6. Conclusions

The user study showed that in physical human-robot interactions, younger and lighter participants generally interacted more efficiently with the robot, with shorter duration of the push and lower impulses, while older and heavier participants tended to require longer and more forceful interactions (i.e., pushing harder and longer). Gender differences also emerged, with men typically applying longer and stronger pushes than women, particularly in error-heavy conditions.

Our findings suggest that both system design (operational mode and error rate) and participant characteristics (age, weight, and gender) shaped user experience and physical interaction with the robot lamp.

Furthermore, the implementation of piezoresistive force sensors as push buttons introduces new opportunities for haptic feedback and user experience design. Due to their minimal plunge travel, these sensors can generate varying sensations depending on the programmed force threshold. For instance, when the activation force is set at a low level, the button feels “soft” or highly responsive, whereas a higher threshold produces the impression of a “hard” or even “faulty” button.

This capability expands the robot’s interaction design beyond a simple two-state system (on/off; “I want” or “I do not want”) into a continuous spectrum of possibilities, ranging from subtle expressions of intent (“I want it”) to more urgent demands (“I want it now!”). In this way, user experience can be dynamically modified through the robot’s customizable characteristics, achieved not by physical hardware changes but by software adjustments and sensor configurations tailored to the robot’s specific interface and intended purpose.

In conclusion, by enabling the interpretation of button press impulse and duration, piezoresistive force sensors become more than weight scales; they become powerful tools for assessing and shaping user experience in human-robot interaction.

## Figures and Tables

**Table 1:** Force sensor Honeywell FSG15N1A’s characterization.

		The Microprocessor Digital Reading Resolution of Analog Piezoresistive Force Sensors			
**Reference Weights**		**Left Button**		**Right Button**	
**In Grams**	**In Newtons**	**Digital Counts**	**Digital Counts per Gram**	**Digital Counts**	**Digital Counts per Gram**
1	0.01	3	3.0	No reading	No reading
2	0.02	6	3.0	No reading	No reading
5	0.05	18	3.6	No reading	No reading
10	0.10	36	3.6	No reading	No reading
20	0.20	74	3.7	12	0.6
50	0.49	193	3.9	147	2.9
100	0.98	380	3.8	300	3.0
150	1.47	543	3.6	450	3.0
200	1.96	707	3.5	643	3.2
250	2.45	870	3.5	820	3.3
500	4.90	1700	3.4	1600	3.2
750	7.35	2467	3.3	2500	3.3
1000	9.80	3400	3.4	3200	3.2
1100	10.78	3750	3.4	3550	3.2

**Table 2:** Omnibus test.

Outcome	Test Effect	Significance	Effect Size (Partial $\eta^{2}$)	95% CI	ϵ (GG)	*p* (GG)
Time	F = 2.13	*p* = 0.081	0.06 (small)	[0, 0.14]	0.47	0.13
Impulse	F = 10.52	*p* < 0.001	0.25 (moderate)	[0.13, 0.36]	0.55	*p* < 0.001
UX	F = 12.80	*p* < 0.001	0.29 (moderate)	[0.17, 0.39]	0.77	*p* < 0.001

**Table 3:** Correlation matrix.

	Time Pushing Buttons	Impulse from Buttons	Self-Reported UX	Age	Weight	Height	Gender_M
Time	1						
Impulse	0.43	1					
UX	−0.13	−0.33	1				
Age	0.13	0.12	−0.10	1			
Weight	0.15	0.07	−0.11	0.17	1		
Height	0.12	0.01	−0.00	0.21	0.75	1	
Gender_M	0.16	0.02	0.01	0.07	0.46	0.71	1

## Data Availability

Data are contained within the article.

## Abbreviations

HRI	Human-Robot Interaction
UX	User Experience
